# New York World’s Fair

**Published:** 1851-10

**Authors:** 


New York World's Fair.—In a recent letter from Dr. Forbes, of St. Louis, he suggests the propriety (should this fair be gotten up) of the Profession, by contribution, offering an extra premium "for the best designs in spittoons, operating table, chair, teeth and every article appertaining to the profession." We would most heartily second this move, and we have no doubt but that a sufficiently large amount might be raised to make a very handsome premium on each article which should take a prize.

These prizes might be fixed by a committee of the profession, in advance of the fair, and we think they should be liberal, particularly that for the best teeth, and the test to which they are subjected most rigid.

We regard a good chair, instrument stand or table, spittoon, etc., of inestimable value; and have never, as yet, seen the whole of these anything like perfect. We hope the profession will think of these suggestions and, when the time comes, act upon them, or give us some suggestions better, which may promote the good of the profession.

